# A novel nomogram model for clinical outcomes of severe subarachnoid hemorrhage patients

**DOI:** 10.3389/fnins.2022.1041548

**Published:** 2022-11-24

**Authors:** Han-Yu Huang, Bin Yuan, Shu-Juan Chen, Yan-ling Han, Xin Zhang, Qing Yu, Qi Wu

**Affiliations:** ^1^Department of Neurosurgery, Jinling Hospital, Nanjing Medical University, Nanjing, Jiangsu, China; ^2^Department of Neurosurgery, The Affiliated Suzhou Hospital of Nanjing Medical University, Suzhou, Jiangsu, China; ^3^Department of Neurosurgery, Jinling Hospital, School of Medicine, Nanjing University, Nanjing, Jiangsu, China; ^4^Department of Clinical Laboratory, Affiliated Hospital of Nanjing University of Chinese Medicine, Nanjing, Jiangsu, China

**Keywords:** aneurysm, subarachnoid hemorrhage, prognosis, nomogram, inflammation

## Abstract

**Background:**

Systemic responses, especially inflammatory responses, after aneurysmal subarachnoid hemorrhage (SAH) are closely related to clinical outcomes. Our study aimed to explore the correlation between the systemic responses in the acute stage and the mid-term outcomes of severe SAH patients (Hunt-Hess grade III-V).

**Materials and methods:**

Severe SAH patients admitted to Jinling Hospital from January 2015 to December 2019 were retrospectively analyzed in the study. The univariate and multivariate logistic regression analyses were used to explore the risk factors of 6-month clinical outcomes in severe SAH patients. A predictive model was established based on those risk factors and was visualized by a nomogram. Then, the predictive nomogram model was validated in another severe SAH patient cohort from January 2020 to January 2022.

**Results:**

A total of 194 patients were enrolled in this study. 123 (63.4%, 123 of 194) patients achieved good clinical outcomes at the 6-month follow-up. Univariate and multivariate logistic regression analysis revealed that age, Hunt-Hess grade, neutrophil-to-lymphocyte ratio (NLR), and complications not related to operations were independent risk factors for unfavorable outcomes at 6-month follow-up. The areas under the curve (AUC) analysis showed that the predictive model based on the above four variables was significantly better than the Hunt-Hess grade (0.812 vs. 0.685, *P* = 0.013). In the validation cohort with 44 severe SAH patients from three different clinical centers, the AUC of the prognostic nomogram model was 0.893.

**Conclusion:**

The predictive nomogram model could be a reliable predictive tool for the outcome of severe SAH patients. Systemic inflammatory responses after SAH and complications not related to operations, especially hydrocephalus, delayed cerebral ischemia, and pneumonia, might be the important risk factors that lead to poor outcomes in severe SAH patients.

## Introduction

Aneurysmal subarachnoid hemorrhage (SAH) accounts for 5% of all strokes and contributes to various subsequent neurological dysfunctions and cognitive impairments, causing social and financial burdens (Etminan et al., 2019; Modi et al., 2019). SAH caused by aneurysm rupture usually results in high mortality and morbidity, especially if the SAH patients present with higher Hunt-Hess grades (Hunt-Hess grade III-V) (Karhunen et al., 2021; Rumalla et al., 2021). Although Hunt-Hess grade (HH) and the World Federation of Neurosurgical Societies grade (WFNS) are widely used in severity assessments of SAH patients, there are still several limitations in outcome prediction. In clinical practice, we found that some patients had presented with poor Hunt-Hess grades after SAH, but the patients could achieve favorable prognoses after targeted treatment. Hence, establishing a predictive model for severe SAH patients that would help the physicians to choose neuroprotective therapies targeting damage mechanisms, and predict outcomes of the patients, remains an urgent concern.

Severe SAH patients usually present with transient or persistent loss of consciousness and different levels of motor dysfunction, often accompanied by systemic complications leading to high mortality or worse functional outcomes (de Oliveira Manoel et al., 2016; Chen et al., 2021). Emergency computed tomography (CT) scans often showed that these patients have suffered from extensive subarachnoid hemorrhage, even with intracerebral or intraventricular hemorrhage, which could lead to acute hydrocephalus and cerebral herniation. Such complicated and serious conditions result in more complications during the perioperative period. Previous studies have developed several tools to predict the clinical outcomes of poor-grade SAH patients (Chen et al., 2021; Shen et al., 2021). However, those predictive models have not been widely accepted. In our study, we screened out variables concerning different aspects of severe SAH patients to establish a reliable predictive nomogram.

## Materials and methods

### Patients

**FIGURE 1:**
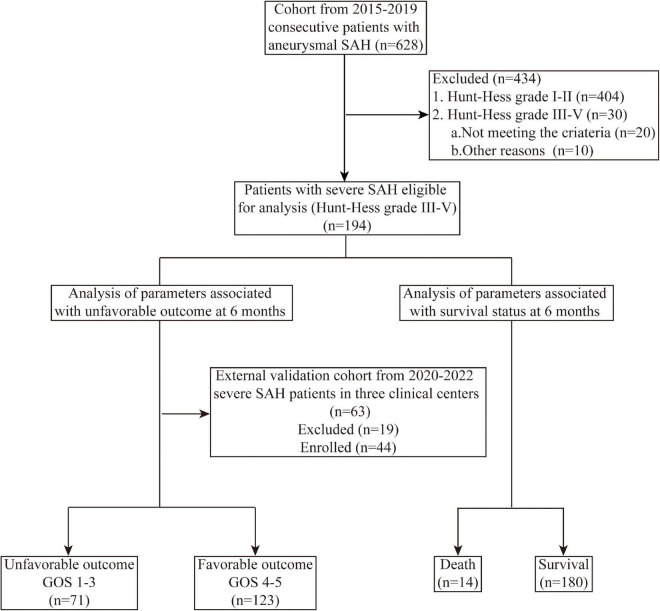
Flowchart of patients’ selection and study process. SAH, subarachnoid hemorrhage; GOS, Glasgow outcome score.

This study was carried out following the Declaration of Helsinki and was approved by the Institutional Review Board of Jinling Hospital. Consecutive severe SAH patients admitted to the Department of Neurosurgery at Jinling Hospital between May 2015 and December 2019 were enrolled in our retrospective study. The severe SAH patients were defined as SAH patients with preoperative poor Hunt-Hess grades (HH III-V) or SAH patients with loss of consciousness. The inclusion criteria of the study were as follows: (1) patients aged ≥ 18; (2) patients diagnosed with SAH by CT and CT angiography (CTA) or digital subtraction angiography (DSA); (3) patients were treated by endovascular coiling; (4) patients that had complete electronic medical records (EMR); and (5) patients had a 6-month follow-up. The study reviewed the EMRs of 628 consecutive patients with aneurysmal SAH. A total of 194 severe SAH patients met the inclusion criteria and were involved in establishing the predictive model. Additionally, the predictive model was validated in an external cohort of 44 severe SAH patients from January 2020 to January 2022, who came from three different clinical centers. The detailed inclusion processes are presented in Figure 1.

### Clinical treatment protocol

Two neurosurgeons independently evaluated the severity of SAH patients according to Hunt-Hess Grade Scale before the surgery. The ruptured aneurysms were coiling within 24 h of the initial rupture. Under general anesthesia, endovascular coiling was transfemorally performed. Systemic anticoagulation with heparin was used before a 6-F guiding catheter was inserted, and it was neutralization with protamine after the endovascular coiling. Stent-assisted coiling or coiling alone was done depending on the characteristics of the aneurysm and the parent artery. In the case of stent-assisted coiling, patients were administered 300 mg aspirin and 300 mg clopidogrel rectally or through a nasogastric tube 2 h before the stent deployment. Dual antiplatelet treatment (100 mg/d aspirin and 75 mg/d clopidogrel) was used for 3 months or longer after the endovascular coiling.

All severe SAH patients were carefully treated after admission. For the management of blood pressure, it was controlled in a stable state (the fluctuation of blood pressure was no more than 10% of the blood pressure at admission) before surgery; after the operation, it was held in a normal range. In case of delayed cerebral ischemia or vasospasm after surgery, the blood pressure shall be increased by 20% based on baseline blood pressure to ensure cerebral perfusion. We routinely administered nimodipine to prevent vasospasm during the peri-operative period. In addition, we gave patients symptomatic treatment, such as proper fluid infusion and volume expansion, proton pump inhibitors to prevent stress ulcers, low-dose mannitol to reduce intracranial pressure, appropriate anti-infection therapy, etc.

### Clinical variables and outcome assessment

Baseline clinical and demographic information such as age, gender, medical history, HH grade, aneurysm number, size, shape, blood test results, and complications were obtained from the EMR. Complications related to operations involved intraoperative hemorrhagic and ischemic events, as well as postoperative cerebral hernia and intracranial infection; complications not related to operations referred to complications caused by the procession of SAH, including hydrocephalus, vasospasm, delayed cerebral ischemia (DCI), pneumonia, gastrointestinal bleeding, and other organ dysfunction.

Functional outcome at 6 months was assessed by three independent neurosurgeons using the Glasgow Outcome Scale (GOS) score *via* neurological examination or telephone interview. GOS scores are classified as good recovery (5), moderate disability (4), severe disability (3), persistent vegetative state (2), and death (1). A GOS score of 4–5 is defined as favorable clinical outcomes, and a GOS score of 1–3 is considered unfavorable clinical outcomes.

### Statistical analysis

Quantitative data are expressed as the mean ± standard deviation and were analyzed by unpaired Student’s *t*-test or the Mann–Whitney U test. Pearson χ2 or Fisher’s exact test was used to categorizing categorical variables reported as counts. Univariate and multivariate logistic regression analysis was performed to explore the independent risk factors for the 6-month unfavorable clinical outcome. A receiver operating characteristic (ROC) curve and areas under the curve (AUC) was used to analyze the accuracy of different predictive models. Statistical analysis was performed using SPSS version 18 software (IBM, Armonk, NY, USA). *P* < 0.05 was considered statistically significant. A nomogram was used to visualize the predictive model using RStudio (R software version 4.0.2). Nomogram discrimination was assessed using the C-index to calculate sensitivity and specificity for prediction at each cut-off value. The C-index represents the AUC of which value assigned 0.5 and 1.0 indicates zero and perfect capability to predict the good prognosis rate of severe aSAH patients. The calibration curve was determined using the Hosmer–Lemeshow test and plotted by RStudio. The decision curve analysis (DCA) was then used to evaluate the clinical net benefit of the novel predictive model.

## Results

### Clinical characteristics in severe aSAH patients

A total of 194 patients diagnosed with severe SAH (HH III-IV) were enrolled as a training cohort. There were 123 patients (63.4%, 123 of 194) with favorable 6-month outcomes, while 71 (36.6%, 71 of 194) suffered from unfavorable outcomes. Thirty-two patients had complications related to operations (16.5%, 32 of 194), and 138 patients had complications not related to operations (69.7%, 138 of 194). Complications related to operations comprised of 21 cases in intracranial infections (10.8%, 21 of 194), 7 cases in cerebral ischemia (3.6%, 7 of 194), 4 cases in cerebral infarction (2.0%, 4 of 194), and 1 for brain herniation (0.5%, 1 of 194) and intracerebral bleeding (0.5%, 1 of 194), respectively. Complications not related to operations contained 99 cases of pneumonia (51.0%, 99 of 194), 43 cases of hydrocephalus (22.2%, 43 of 194), 33 cases of vasospasm (17.0%, 33 of 194), 9 cases of DCI (4.6%, 9 of 194), 8 cases of gastrointestinal bleeding (4.1%, 8 of 194).

### Risk factors for 6-month clinical outcomes

As shown in Table 1, compared with the favorable outcome patients, patients with unfavorable outcomes had significantly higher age (61.2 ± 12.9 vs. 57.0 ± 10.6, *P* = 0.014) and HH (HH III 22 vs. 78, HH III 42 vs. 40, HH IV 9 vs. 5, *P* < 0.001). In terms of underlying diseases, hypertension (44.7% vs. 28.2%, *P* = 0.023) was more frequently observed in patients with unfavorable outcomes. Moreover, severe SAH patients with poor prognoses presented more complications not related to operations (93% vs. 58.5%, *P* < 0.001). Reviewing the records of peripheral blood routine examination, we found that patients with poor outcomes had higher levels of C-reactive protein (CRP) (27.63 ± 42.61 vs. 16.35 ± 28.34, *P* = 0.024), neutrophil-to-lymphocyte ratio (NLR) (19.51 ± 17.97 vs. 14.76 ± 8.85, *P* = 0.014), whereas the level of direct bilirubin was significantly lower in those patients (0.82 ± 3.02 vs. 1.39 ± 2.68, *P* = 0.027). Then, multivariate logistic regression analysis showed that age (odds ratio (OR), 1.038; 95% confidence interval (95% CI), 1.006 – 1.071; *P* = 0.018), HH (OR, 2.331; 95% CI, 1.312–4.142; *P* = 0.004), complications not related to operations (OR, 5.795; 95% CI, 2.064–16.271; *P* < 0.001) and NLR (OR, 1.033; 95% CI, 1.002–1.065; *P* = 0.034) were independent risk factors for 6-month unfavorable clinical outcome in severe SAH patients.

**TABLE 1:** Potential risk factors related to 6-month unfavorable outcomes in severe subarachnoid hemorrhage (SAH) patients.

Variables	Favorable outcome (*n* = 123)	Unfavorable outcome (*n* = 71)	*P*-value	Multivariate logistic regression	
				OR [95% CI]	*P*-value
Gender			0.659		
Men	48	30			
Women	75	41			
Ages (year)	57.0 ± 10.6	61.2 ± 12.9	0.014*	1.038 [1.006, 1.071]	**0.018**
Aneurysm number			0.533		
Single	94	57			
Multiple	29	14			
Aneurysm shape			0.686		
Saccular	103	61			
Non-saccular	20	10			
Hunt-Hess grade			**<0.001**	2.331 [1.312, 4.142]	**0.004**
III	78	20			
IV	40	42			
V	5	9			
Aneurysm size (mm)	5.52 ± 3.19	5.59 ± 4.64	0.642^#^		
Treatment			0.598		
Coiling	61	38			
Assisted coiling	62	33			
Hypertension			**0.023**	0.545 [0.261, 1.136]	0.105
Yes	55	20			
No	68	51			
Diabetes mellitus			0.541		
Yes	9	3			
No	114	68			
Heart disease			>0.999		
Yes	4	2			
No	119	69			
Cerebrovascular disease			0.361		
Yes	6	6			
No	117	65			
Complications related to operations			0.187		
Yes	17	15			
No	106	56			
Complications not related to operations			**<0.001**	5.795 [2.064, 16.271]	**<0.001**
Yes	72	66			
No	51	5			
Hemoglobin (g/L)	132.78 ± 17.65	135.65 ± 16.83	0.269*		
Red blood cell count (×10^12^/L)	4.43 ± 0.50	4.53 ± 0.59	0.225*		
White blood cell count (×10^9^/L)	14.67 ± 5.12	15.71 ± 5.32	0.183*		
Hematocrit (L/L)	0.396 ± 0.049	0.405 ± 0.048	0.204*		
Lymphocyte count (×10^9^/L)	1.13 ± 0.73	1.24 ± 1.33	0.324^#^		
Neutrophil count (×10^9^/L)	12.81 ± 4.65	13.71 ± 5.22	0.213*		
Monocyte count (×10^9^/L)	0.73 ± 0.64	0.69 ± 0.46	0.867^#^		
Basophil count (×10^9^/L)	0.02 ± 0.02	0.020 ± 0.02	0.838^#^		
Eosinophil count (×10^9^/L)	0.02 ± 0.04	0.03 ± 0.06	0.242^#^		
Mean red blood cell volume (fL)	89.46 ± 6.26	89.97 ± 5.25	0.564^#^		
Mean red blood cell hemoglobin (pg)	29.97 ± 2.38	30.05 ± 2.03	0.509^#^		
Mean corpuscular hemoglobin content ratio (g/L)	334.85 ± 11.89	329.80 ± 37.57	0.617^#^		
Red cell volume distribution width (%)	13.29 ± 1.97	13.02 ± 0.96	0.531^#^		
Platelet (×10^9^/L)	197.48 ± 63.08	208.92 ± 73.96	0.241^#^		
Plateletcrit (%)	0.204 ± 0.056	0.208 ± 0.066	0.477^#^		
Mean platelet volume (fL)	10.55 ± 1.58	10.10 ± 1.42	0.052*		
C-reactive protein (mg/L)	16.35 ± 28.34	27.63 ± 42.61	**0.024** ^#^	1.005 [0.996, 1.015]	0.273
Neutrophil/Lymphocyte ratio	14.76 ± 8.85	19.51 ± 17.97	0.014*	1.033 [1.002, 1.065]	**0.034**
Platelet/Lymphocyte ratio	222.23 ± 120.95	271.15 ± 220.74	0.206^#^		
Lymphocyte/Monocyte ratio	2.05 ± 1.48	12.73 ± 88.97	0.464^#^		
Total protein (g/L)	71.89 ± 7.87	72.61 ± 8.46	0.553*		
Albumin (g/L)	40.95 ± 5.31	41.32 ± 6.05	0.654*		
BUN (mmol/L)	5.56 ± 3.22	5.30 ± 2.27	0.726^#^		
Creatinine (μmol/L)	68.64 ± 77.69	59.25 ± 32.05	0.909^#^		
K^+^ (mmol/L)	3.52 ± 0.47	3.55 ± 0.44	0.643^#^		
Na^+^ (mmol/L)	138.08 ± 12.05	139.66 ± 5.08	0.218^#^		
Cl^–^ (mmol/L)	101.82 ± 4.51	101.70 ± 6.24	0.899^#^		
Total bilirubin (mmol/L)	15.39 ± 8.30	15.58 ± 9.23	0.917^#^		
Direct bilirubin (mmol/L)	1.39 ± 2.68	0.82 ± 3.02	**0.027** ^#^		
Indirect bilirubin (mmol/L)	9.83 ± 7.20	9.53 ± 7.41	0.548^#^		
Uric acid (mmol/L)	272.91 ± 134.81	254.76 ± 97.61	0.640^#^		
Glucose (mmol/L)	8.89 ± 2.89	9.38 ± 2.67	0.106^#^		

*According to the *t*-test. ^#^According to the Mann–Whitney U test. OR, odds ratio; 95% CI, 95% confidence interval.

The bold values mean the *p*-value that is statistically significant (<0.05).

To further determine the correlation between different complications not related to operations and 6-month unfavorable outcomes, we analyzed them by univariate logistic regression. The results were showed in Table 2 that the presence of hydrocephalus (OR, 2.795; 95% CI, 1.397–5.592; *P* = 0.004), DCI (OR, 6.617; 95% CI, 1.335–32.788; *P* = 0.021), and pneumonia (OR, 6.172; 95% CI, 3.165–12.034; *P* < 0.001) were significantly related to the 6-month unfavorable outcome.

**TABLE 2:** Potential risk factors related to 6-month survival in severe SAH patients.

Variables	Survival (*n* = 180)	Death (*n* = 14)	*P*-value	Multivariate logistic regression	
				OR [95% CI]	*P*-value
Gender			0.438		
Men	71	7			
Women	109	7			
Ages (year)	59.5 ± 11.7	59.5 ± 14.1	0.782*		
Aneurysm number			0.199		
Single	142	9			
Multiple	38	5			
Aneurysm shape			0.458		
Saccular	153	11			
Non-saccular	27	3			
Hunt-Hess grade			0.087		
III	93	5			
IV	73	6			
V	11	3			
Aneurysm size (mm)	5.67 ± 4.01	4.97 ± 2.15	0.760^#^		
Treatment			0.525		
Coiling	93	6			
Assisted coiling	87	8			
Hypertension			0.421		
Yes	71	4			
No	109	10			
Diabetes mellitus			0.604		
Yes	11	1			
No	169	13			
Heart disease			>0.999		
Yes	6	0			
No	174	14			
Cerebrovascular disease			0.210		
Yes	10	2			
No	170	12			
Complications related to operations			0.254		
Yes	28	4			
No	152	10			
Complications not related to operations			0.358		
Yes	126	12			
No	54	2			
Hemoglobin (g/L)	133.94 ± 16.70	132.36 ± 25.18	0.743*		
Red blood cell count (×10^12^/L)	4.46 ± 0.51	4.52 ± 0.86	0.660*		
White blood cell count (×10^9^/L)	15.07 ± 5.32	14.81 ± 3.54	0.782^#^		
Hematocrit (L/L)	0.399 ± 0.047	0.396 ± 0.067	0.824*		
Lymphocyte count (×10^9^/L)	1.16 ± 0.87	1.36 ± 2.04	0.317^#^		
Neutrophil count (×10^9^/L)	13.17 ± 4.94	12.76 ± 4.04	0.898^#^		
Monocyte count (×10^9^/L)	0.72 ± 0.58	0.63 ± 0.51	0.328^#^		
Basophil count (×10^9^/L)	0.02 ± 0.02	0.02 ± 0.02	0.728^#^		
Eosinophil count (×10^9^/L)	0.02 ± 0.04	0.04 ± 0.09	0.865^#^		
Mean red blood cell volume (fL)	89.76 ± 5.81	88.19 ± 7.01	0.508^#^		
Mean red blood cell hemoglobin (pg)	30.04 ± 2.20	29.40 ± 2.90	0.380^#^		
Mean corpuscular hemoglobin content ratio (g/L)	333.01 ± 25.22	330.00 ± 16.05	0.900^#^		
Red cell volume distribution width (%)	13.20 ± 1.69	13.06 ± 1.45	0.236^#^		
Platelet (× 10^9^/L)	200.68 ± 63.10	214.29 ± 110.66	0.876^#^		
Plateletcrit (%)	0.205 ± 0.057	0.213 ± 0.060	0.829^#^		
Mean platelet volume (fL)	10.39 ± 1.54	10.28 ± 1.56	0.707^#^		
C-reactive protein (mg/L)	20.10 ± 35.14	25.30 ± 26.82	0.106^#^		
Neutrophil/Lymphocyte ratio	16.21 ± 13.02	20.20 ± 14.16	0.384^#^		
Platelet/Lymphocyte ratio	233.41 ± 147345	326.69 ± 316.02	0.611^#^		
Lymphocyte/Monocyte ratio	6.22 ± 55.90	2.56 ± 2.86	0.853^#^		
Total protein (g/L)	72.53 ± 7.76	67.38 ± 10.61	**0.023** ^#^	**1.081 [1.010, 1.156]**	**0.025**
Albumin (g/L)	41.32 ± 5.34	38.05 ± 7.71	0.034*		
BUN (mmol/L)	5.37 ± 2.81	6.62 ± 3.86	0.382^#^		
Creatinine (μmol/L)	64.30 ± 64.80	76.87 ± 64.00	0.726^#^		
K^+^ (mmol/L)	3.51 ± 0.45	3.75 ± 0.51	0.117^#^		
Na^+^ (mmol/L)	138.55 ± 10.42	140.10 ± 3.61	0.665^#^		
Cl^–^ (mmol/L)	101.62 ± 5.21	103.79 ± 4.79	0.050^#^		
Total bilirubin (mmol/L)	15.65 ± 8.72	13.08 ± 7.30	0.204^#^		
Direct bilirubin (mmol/L)	1.25 ± 2.89	0.36 ± 1.36	0.187^#^		
Indirect bilirubin (mmol/L)	9.97 ± 7.32	6.63 ± 5.77	**0.037** ^#^		
Uric acid (mmol/L)	265.21 ± 124.55	278.71 ± 93.82	0.448^#^		
Glucose (mmol/L)	8.97 ± 2.75	10.40 ± 3.45	0.093^#^		
Na^+^/K^+^ ratio	40.05 ± 5.88	38.00 ± 5.30	0.172^#^		
Glucose/K^+^ ratio	2.50 ± 1.16	2.60 ± 1.16	0.739^#^		

*According to the *t*-test. ^#^According to the Mann–Whitney U test. OR, odds ratio; 95% CI, 95% confidence interval.

The bold values mean the *p*-value that is statistically significant (<0.05).

In addition, further analysis showed in Table 3 that lower levels of serum total protein (67.38 ± 10.61 vs. 72.53 ± 7.76, *P* = 0.023), albumin (38.05 ± 7.71 vs. 41.32 ± 5.34, *P* = 0.034), and indirect bilirubin (6.63 ± 5.77 vs. 9.97 ± 7.32, *P* = 0.037) were observed in dead SAH patients at 6-month follow-up. Furthermore, multivariate logistic regression analysis revealed that low serum total protein was an independent risk factor for 6-month death.

**TABLE 3:** Subgroup analysis of complications not related to operations.

Complications not related to operations	Univariate logistic regression	
	OR [95% CI]	*P*-value
Hydrocephalus	2.795 [1.397, 5.592]	**0.004**
Cerebral herniation	3.312E9 [0, 3.312E9]	0.999
Vasospasm	1.344 [0.627, 2.881]	0.447
Pneumonia	6.172 [3.165, 12.034]	**<0.001**
Delayed cerebral ischemia	6.617 [1.335, 32.788]	**0.021**
Gastrointestinal bleeding	0.565 [0.111, 2.878]	0.492

The bold values mean the *p*-value that is statistically significant (<0.05).

### The establishment of a predictive model for 6-month clinical outcomes

**FIGURE 2:**
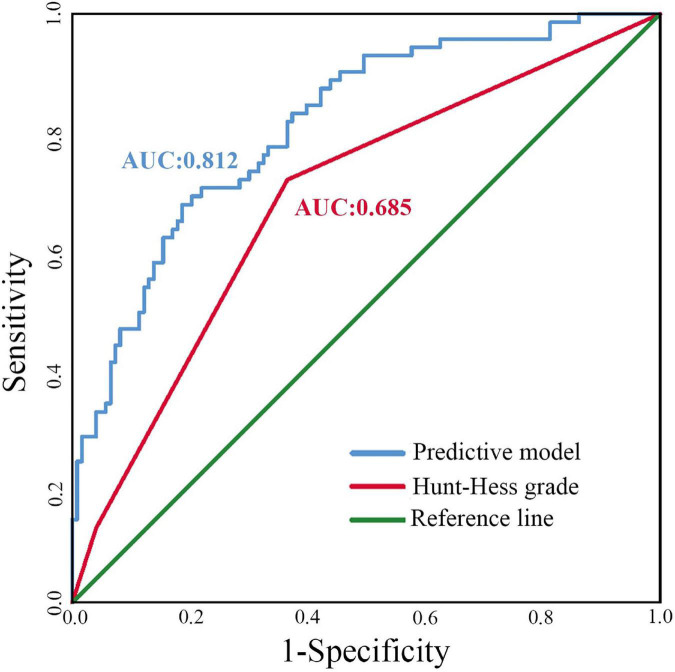
The ROC curves for the different predictive models. AUC, area under the curve.

In the present study, we constructed a predictive model for a 6-month clinical outcome based on the above risk factors, including age, HH, NLR, hydrocephalus, DCI, and pneumonia.^1^ ROC curve analysis showed the AUC of the constructed predictive model was 0.812 (95% CI 0.750–0.873, *P* < 0.001). Moreover, the sensitivity and specificity of the predictive model were 77.2 and 71.8%. Compared with the AUC value of HH grade, the constructed predictive model performed better in predicting the 6-month clinical outcomes of severe SAH patients (0.812 vs. 0.685, *Z* = 2.493, *P* = 0.013) (Figure 2).

**FIGURE 3:**
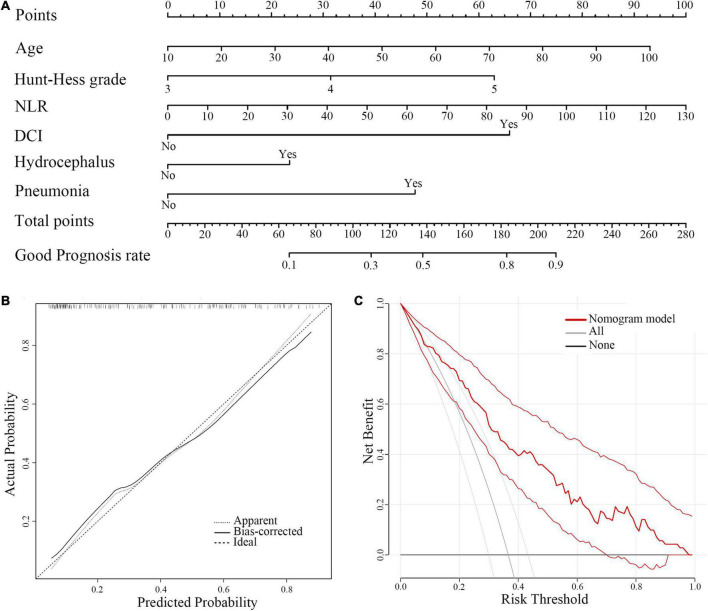
A Nomogram model for predicting 6-month favorable outcomes in severe SAH patients (HH III-IV). **(A)** A predictive nomogram model. **(B)** The calibration curves for the nomogram. **(C)** The DCA of the training cohort. NLR, neutrophil-to-lymphocyte ratio; DCI, delayed cerebral ischemia; DCA, decision curve analysis.

For visualization and convenient clinical usage of the predictive model, as shown in Figure 3, a nomogram was built incorporating these six factors. The calibration plot showed good consistency (*B* = 40 repetitions, mean absolute error = 0.026, *n* = 194) between the prediction by nomogram and actual observation. Decision curve analysis (DCA) also revealed that the nomogram conferred more benefit in the training cohort.

### The validation of the predictive nomogram

A total of 44 severe SAH patients from three different interventional neuroradiology centers dated from January 2020 to January 2022 were included in the validation cohort. There were 32 (72.7%, 32 of 44) patients with favorable outcomes, while 12 (27.2%, 12 of 44) patients suffered from unfavorable outcomes. Complications not related to operations were observed in 31 patients (70.5%, 31 of 44), and complications related to operations were seen in 11 patients (25%, 11 of 44). For further details, 26 cases had pneumonia, 10 cases had hydrocephalus, 8 had DCI, 3 had vasospasm, and 2 had gastrointestinal bleeding. In addition, there was 1 case of pulmonary edema, heart failure, and chronic subdural hematoma.

**FIGURE 4:**
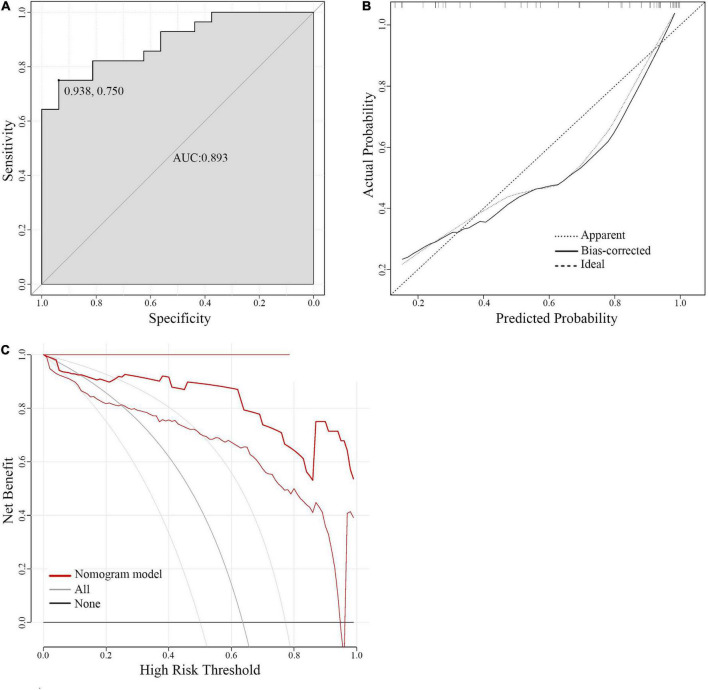
The calibration plots and decision curve analysis of predictive nomogram in the validation cohort. **(A)** The ROC curve of the validation cohort. **(B)** The calibration curve for the validation cohort. **(C)** DCA of the validation cohort. ROC curve, receiver operating characteristic curve; DCA, decision curve analysis.

Complications related to operations included 7 cases of cerebral ischemia (15.9%, 7 of 44), 6 cases of intracranial infection (13.6%, 6 of 44), 3 cases of intracerebral bleeding (6.8%, 3 of 44), and 1 case of cerebral infarction (2.2%, 1 of 44). The stability and reliability of the predictive nomogram were validated in a new cohort of 44 severe SAH patients. The ROC curve analysis showed that the nomogram had a good predictive ability with an AUC value of 0.893 (Figure 4). The calibration plot (*B* = 40 repetitions, mean absolute error = 0.065, *n* = 44) and DCA also indicated that the predictive nomogram was stable and reliable.

## Discussion

Multiple predictive models have been established to estimate the prognosis of SAH patients (Jaja et al., 2018; Mourelo-Farina et al., 2021). However, the predictive models for poor-grade SAH patients are rarely reported. Although much literature has revealed that a series of factors were significantly related to poor outcomes, few studies constructed a predictive model to predict the prognosis of poor-grade SAH patients (summarized in Table 4). Previous studies suggested that age, modified Fisher grade, DCI, hydrocephalus, and pupil reactivity were significantly correlated with poor clinical outcomes of poor-grade SAH patients (Ironside et al., 2019; Ota et al., 2019; Wang et al., 2019; Zheng et al., 2019; Liu et al., 2020; Yoshikawa et al., 2020; Shen et al., 2021). Liu et al. retrospectively analyzed 266 patients (130 clipping and 136 coiling) with WFNS IV-V from multi-center and found that older age, absence of pupillary reactivity, lower Glasgow coma score (GCS), and higher modified Fisher grade were independent predictors of 12-month unfavorable outcome (Liu et al., 2020). Then, they built a decision tree model including the above predictors with an accuracy of 0.833. However, this model was constructed with more subjective markers such as pupil reflex and GCS, which primarily reflected the patient’s status at onset instead of the general condition of patients. Additionally, this model did not consider the complications following SAH that significantly affected the clinical outcomes. Another predictive model recently reported by Shen et al. has included the complications that occurred in the process of SAH. They enrolled 147 patients (81 clipping, 43 coiling, and 23 conservative treatment) with WFNS IV-V and established a scoring system based on six independent risk factors (Shen et al., 2021). The accuracy of the scoring system was similar to Liu et al.’s model (0.844 vs.0.833). However, most patients analyzed in their study were treated by clipping, which may bias the results.

**TABLE 4:** A summary of studies on the significant predictors for poor outcomes in poor-grade SAH patients (WFNS IV-V).

References	Patients	Follow-up (months)	Significant predictors for poor outcomes	Predictive model	
				Training cohort	Validation cohort
Ota et al., 2019	186 clipping	12	Age > 75, mindline shift, CSF density(cortical), CSF (ambient cistern)		
Zheng et al., 2019	130 clipping vs. 136 coiling vs. 58 conservative treatment	12	Age, GCS, pupil reactivity, mFisher, conservative treatment	AUC:0.86	
Ironside et al., 2019	65 clipping vs. 71 coiling vs. 3 conservative treatment	6	Age, HH, hydrocephalus, EVD, CSF shunt		
Wang et al., 2019	71 clipping vs. 33 coiling	6	Fisher, low density CT, hydrocephalus, coiling		
Liu et al., 2020	130 clipping vs. 136 coiling	12	Age, pupil reactivity, GCS, mFisher	AUC:0.833	AUC:0.895
Yoshikawa et al., 2020	64 clipping vs. 54 coiling	3,12	Fisher, ICH, DCI, plasma D-dimer, sodium		
Shen et al., 2021	81 clipping vs. 43 coiling vs. 23 conservative treatment	6	Age > 65, therapeutic strategy, WFNS(V), DCI, SDH, cerebral herniation	AUC:0.844	AUC:0.831
Imberti et al., 2021	99 coiling	3	ICP, CPP		

HH, Hunt-Hess grade; CSF, cerebrospinal fluid; EVD, extraventricular drainage; CT, computed tomography; GCS, Glasgow coma scale; mFisher, modified Fisher score;, ICH, intracranial hemorrhage; DCI, delayed cerebral ischemia; SDH, subdural hematoma; ICP, intracranial pressure; CPP, cerebral perfusion pressure; AUC, area under the curve.

The previous study has suggested that loss of consciousness at the ictus of SAH was associated with worse clinical grades and poor outcomes (Macdonald, 2016). Moreover, Suwatcharangkoon et al. also reported that loss of consciousness is an important marker of early brain injury after SAH and a predictor of 12-month death or poor prognosis (Hendrix et al., 2020). Thus, we reviewed the EMRs of severe SAH patients with HH III-V, SAH patients with loss of consciousness, and retrospectively analyzed the risk factors for 6-month unfavorable outcomes. Then, a predictive model, including age, HH, NLR, hydrocephalus, DCI, and pneumonia, was established to predict the 6-month clinical outcomes of severe SAH patients. Our predictive model is comprehensive, not only considering the primary brain injury (e.g., HH), but also considering the secondary complications following SAH (e.g., hydrocephalus, DCI, and pneumonia). Additionally, the predictive model was built based on coiling patients, which avoids the collateral damage from surgical clipping affecting the functional outcome of SAH. To some extent, the study is the first to establish a reliable predictive tool for long-term functional outcome prediction of severe SAH patients to improve informed decision-making. In our study, the established predictive model was presented with a nomogram. Compared with the reported predictive models, the nomogram prevails in its accuracy in probability prediction and simplistic visual style. It can provide many conveniences for clinicians to make decisions. We have also provided a dynamic version of a nomogram based on the web page so that you can quickly get the predicted results on your mobile device anytime, anywhere (see text footnote 1). This useful tool can help us formulate more individualized treatments and better prognostic prediction.

Our nomogram performed better in predicting the 6-month clinical outcomes of severe SAH patients compared with a single HH grade (AUC 0.812 vs. 0.685, *P* < 0.05). The predictive model containing a single variable cannot commendably predict the prognosis of severe SAH patients with complicated conditions. The HH and WFNS are effective and time-honored grading systems evaluating the severity of SAH and primary brain injury (Tewari et al., 2015). In clinical practice, they are often used to assess the prognosis of SAH patients roughly. However, HH or WFNS alone is not accurate enough to predict the outcomes of severe SAH patients with complicated conditions. There is increasing evidence that secondary injury after SAH is an unneglectable factor for patients’ prognosis (Sharma et al., 2021; Yun et al., 2021). Hence, the multivariate predictive models, including HH or WFNS, presented a better advantage in prognostication, which is also reflected in our study.

In the present study, we also found for the first time that NLR is significantly related to the poor prognosis of patients with severe SAH and included it in the final nomogram model. Recently, studies on the diagnostic value of NLR in subarachnoid hemorrhage have gradually increased (Giede-Jeppe et al., 2019). An elevated NLR has been proven to be related to poor prognosis and secondary complications such as rebleeding, delayed cerebral ischemia, and postoperative pneumonia (Wu et al., 2019; Wang et al., 2020). However, the significance of NLR in the prognosis of severe SAH patients remains unveiled, and our study is the first to report that the increased NLR is associated with long-term outcomes in severe SAH patients. The inflammatory response following SAH was closely correlated with the severity of SAH and significantly contributed to poor outcomes. NLR could be a reliable marker that reflects the systemic inflammatory status.

Excessively elevated neutrophils may inhibit the adaptive immune system and suppress T-cell activation, proliferation, and their effector functions. Furthermore, neutrophils may interact with the activated endothelial cells (ECs), aggravating inflammatory responses, therefore leading to the destruction of the blood-brain barrier (BBB), brain swelling, and ultimately neuronal injury (Maestrini et al., 2020) Tregs. Decreased numbers of lymphocytes were detected in the SAH patients, especially Tregs (Klein and Hunter, 2017). Tregs promote the polarization and transformation of the M2 microglia, which can inhibit the neutrophil-derived MMP-9 and protect the integrity of ECs and BBB (Yang et al., 2020). Therefore, NLR can exhaustively reflect the immune and inflammatory response status. Moreover, combined with NLR, the accuracy of HH improved in predicting the prognosis of severe SAH patients (Supplementary Figure 1).

Interestingly, our study found that complications not related to operations at the early stage were also an independent risk factor for 6-month unfavorable outcomes in severe SAH patients. Severe SAH patients often suffer more complications not related to operations, such as hydrocephalus, vasospasm, delayed cerebral ischemia, pneumonia, gastrointestinal bleeding, etc. These complications not related to operations significantly affected the clinical outcome of SAH patients (Chen et al., 2017; Wang and Huang, 2017; Savarraj et al., 2021). Further analysis showed hydrocephalus, DCI, and pneumonia significantly contributed to the 6-month unfavorable outcomes of severe SAH patients. Perioperative complications not related to operations should not be ignored when predicting the prognosis. Hence, our predictive nomogram contained the above three complications. In addition, we also found that severe SAH patients who died at 6-month follow-up characterized low serum total protein and albumin. The nutritional status at admission seems to be positively related to the clinical outcome of severe SAH patients.

Our predictive nomogram also has some disadvantages. Firstly, the nomogram may not be suitable for predicting the prognosis of patients with craniotomy aneurysmal clipping. Secondly, the evaluation system included in the nomogram is Hunt-Hess grade rather than WFNS grade due to the limitations of EMRs, making the evaluation of SAH severity less objective. Finally, the follow-up in our model is 6 months. If the follow-up can be increased up to 12 months or more, the validity and accuracy of the nomogram could be improved.

In conclusion, the nomogram established in this study can reliably predict the long-term prognosis of patients with severe SAH. Six risk factors included in the nomogram can also thoroughly illustrate the pathophysiological characteristics in the early stage of SAH, making the predictive model in our study more reliable. Meanwhile, indicators such as NLR, pneumonia and low serum protein level, etc., identified that patients accompanied with an aggressive inflammatory response and poor nutritional status in the early stage of SAH seem not to achieve an excellent functional outcome, suggesting that we should interfere with inflammation and nutritional condition at the early stage of SAH, and reduce the perioperative complications not related to operations to benefit the patients for better prognosis.

### Limitation

A few limitations should be mentioned. (1) Our study is limited by its retrospective and non-randomized nature. A low volume of cases from a single center may bias our study and undermine the strength of the conclusions. Multicenter, prospective, large cohort clinical studies were needed to refine our study in the hope of helping to differentiate severe SAH patients into those who should be treated and those who should be palliated. (2) Compared with the better-validated WFNS, the only HH grading used in our study seems to be more subjective in the severity assessment of SAH patients, which could contribute to a higher grading of the patients. (3) Incomplete information from EMR leads us to miss potential risk factors. Our study did not include variables such as coagulation function (e.g., prothrombin time, thrombin time, activated partial thromboplastin time, and international normalized ratio) or liver function (e.g., alanine aminotransferase, aspartate aminotransferase). (4) CSF-based biomarkers should be assessed in severe SAH patients. CSF, the circulating fluid in the central nervous system, can provide important information about brain homeostasis. Analyzing the CSF of severe SAH patients can objectively assess the severity of brain injury.

## Conclusion

Our study appeared to be the first to explore the factors associated with 6-month clinical outcomes in severe SAH patients. We identified that (1) age, HH grade, NLR, and complications not related to operations are independent risk factors for 6-month unfavorable outcomes in severe SAH patients; (2) serum total protein level at admission is an independent predictor of 6-month mortality; and (3) a nomogram involving age, HH grade, NLR, hydrocephalus, DCI, and pneumonia is a novel and reliable tool for predicting 6-month clinical outcomes in severe SAH patients. Systemic inflammatory responses after SAH and complications not related to operations, especially hydrocephalus, delayed cerebral ischemia, and pneumonia, might be the important risk factors that lead to poor outcomes in severe SAH patients.

## Data availability statement

The raw data of the article can be available from the authors upon request.

## Ethics statement

This study was approved by the Institutional Review Board of Jinling Hospital. The patients have signed informed consent to participate in the study.

## Author contributions

QW and QY: study concepts, study design, and manuscript review. S-JC and XZ: data collection. Y-LH and BY: statistical analysis. H-YH and BY: manuscript drafting. All authors contributed to the article and approved the submitted version.

## Footnotes

1
https://sahoutcomeprediction.shinyapps.io/severeSAHprediction/

## Supplementary material

Supplementary Figure 1 The ROC curves for the models. NLR, neutrophil-to-lymphocyte ratio.
