# Supplementary figures and images for: A novel nomogram model for clinical outcomes of severe subarachnoid hemorrhage patients
Source: Front Neurosci. 2022 Nov 24;16:1041548. doi: 10.3389/fnins.2022.1041548 (PMC9729550; doi:10.3389/fnins.2022.1041548)

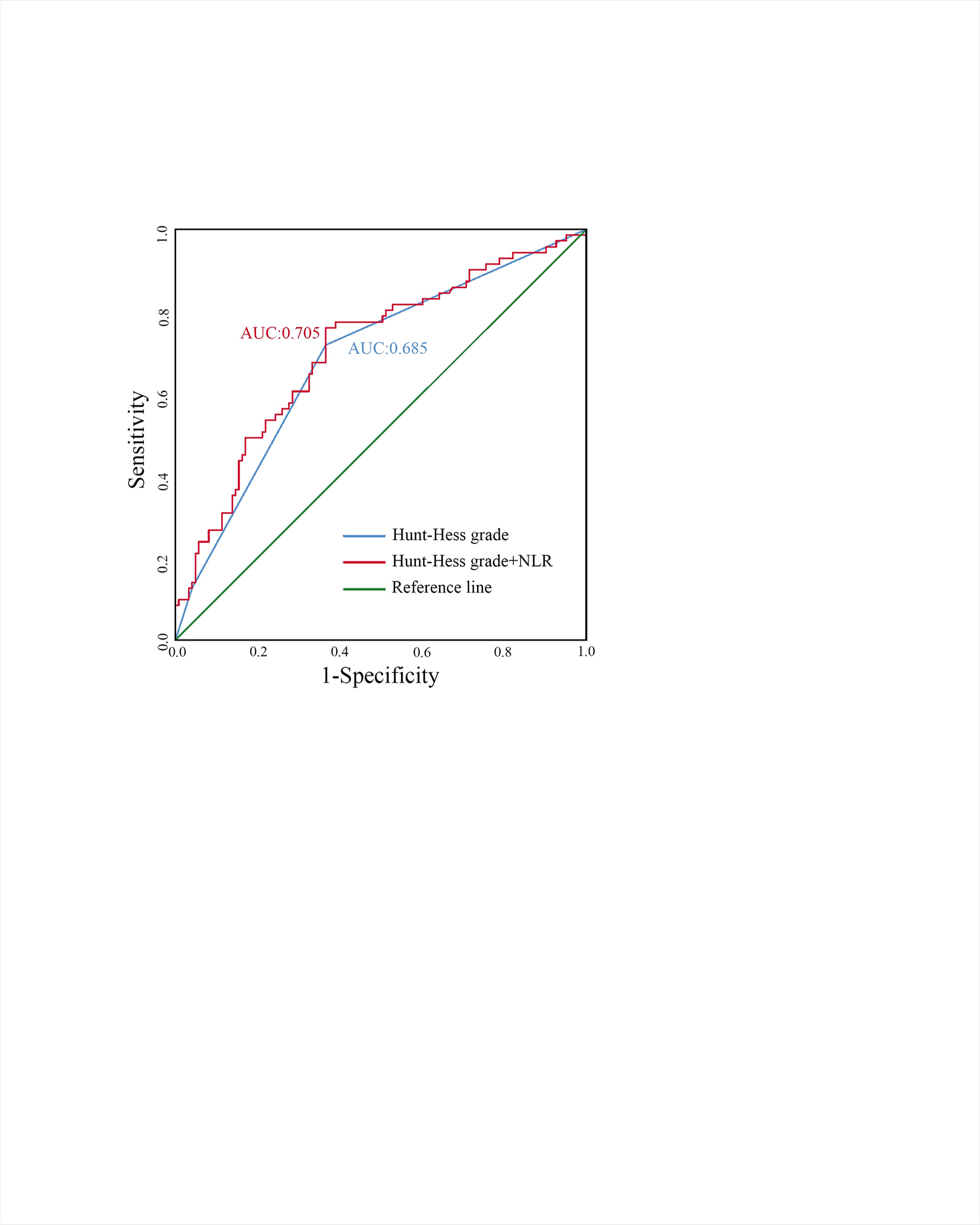

Supplement: Supplementary Figure 1 — The ROC curves for the models. NLR, neutrophil-to-lymphocyte ratio. [file Image_1.tif]
